# MXene/Ag_2_CrO_4_ Nanocomposite as Supercapacitors Electrode

**DOI:** 10.3390/ma14206008

**Published:** 2021-10-12

**Authors:** Tahira Yaqoob, Malika Rani, Arshad Mahmood, Rubia Shafique, Safia Khan, Naveed Kausar Janjua, Aqeel Ahmad Shah, Awais Ahmad, Abdullah A. Al-Kahtani

**Affiliations:** 1 Department of Physics, The Women University Multan, Multan 66000, Pakistan; tahiraroy123@gmail.com (T.Y.); rubiashafique@yahoo.com (R.S.); 2 National Institute of Lasers and Optronics (NILOP), College PIEAS, NILORE, Islamabad 45650, Pakistan; drarshadjanjua@gmail.com; 3 Department of Chemistry, Quaid-I-Azam University, Islamabad 45320, Pakistan; safiakhan715@gmail.com (S.K.); nkausarjanjua@yahoo.com (N.K.J.); 4 Department of Metallurgical Engineering, NED University of Engineering and Technology, Karachi 75270, Pakistan; gr8muet@gmail.com; 5 Departamento de Quimica Organica, Universidad de Cordoba, Edificio Marie Curie (C-3), Ctra Nnal IV-A, Km 396, E14014 Cordoba, Spain; awaisahmad@gcuf.edu.pk; 6 Chemistry Department, College of Science, King Saud University, Riyadh 11451, Saudi Arabia; akahtani@ksu.edu.sa

**Keywords:** MXene nanocomposite, spinel chromite, energy storage, supercapacitors electrodes

## Abstract

MXene/Ag_2_CrO_4_ nanocomposite was synthesized effectively by means of superficial low-cost co-precipitation technique in order to inspect its capacitive storage potential for supercapacitors. MXene was etched from MAX powder and Ag_2_CrO_4_ spinel was synthesized by an easy sol-gel scheme. X-Ray diffraction (XRD) revealed an addition in inter-planar spacing from 4.7 Å to 6.2 Å while Ag_2_CrO_4_ nanoparticles diffused in form of clusters over MXene layers that had been explored by scanning electron microscopy (SEM). Energy dispersive X-Ray (EDX) demonstrated the elemental analysis. Raman spectroscopy opens the gap between bonding structure of as-synthesized nanocomposite. From photoluminence (PL) spectra the energy band gap value 3.86 eV was estimated. Electrode properties were characterized by applying electrochemical observations such as cyclic voltammetry along with electrochemical impedance spectroscopy (EIS) for understanding redox mechanism and electron transfer rate constant K_app_. Additionally, this novel work will be an assessment to analyze the capacitive behavior of electrode in different electrolytes such as in acidic of 0.1 M H_2_SO_4_ has specific capacitance C_sp_ = 525 F/g at 10 mVs^−1^ and much low value in basic of 1 M KOH electrolyte. This paper reflects the novel synthesis and applications of MXene/Ag_2_CrO_4_ nanocomposite electrode fabrication in energy storage devices such as supercapacitors.

## 1. Introduction

The stipulate for well-groomed energy storage strategies is on the hit list in the current state of affairs. To overcome this worldwide issue, supercapacitors were used to pile up energy in electronic applications to store charge, depending upon electrochemical reactions enclosed by them [1]. Narrative layered two-dimensional (2D) material i.e., MXene (Ti_3_C_2_T_x_) comprehensively deliberated to construct electrodes for supercapacitors owing to their high metallic conduction rate and reactive hydrophilic surface. In spite of all the assorted dilemmas including re-crushing and oxidizing of titanium which obstruct Ti_3_C_2_T_x_ to achieve the significant capacitance, cheap carbon electrode material for instance Ti_3_C_2_T_x_, a type of MXene, participated in great technological research for development of supercapacitors electrodes by defeating these issues [2,3,4]. In composite form, Ti_3_C_2_T_x_ deals with the above-mentioned problems due to its excellent specific capacity with lower resistance, significant surface area and the redox active nature of surface functional groups. Supercapacitors (SCs) had been discussed as a promising energy storage tool due to the fast charge/discharge process, high power density in many new technologies and the use of the redox (pseudo-capacitive) mechanism on surface which could be employed for storing more energy than batteries [5,6,7]. The electrochemical capacitors (ECs) mentioned as supercapacitors are taken as the key technology for the promotion of the immense progress in 2D transition metal carbides/nitrides, known as “MXene”. Hence, MXene (Ti_3_C_2_T_x_) showed potential as electrode resource of supercapacitors due to their key factors of intercalation, pseudocapacitance mechanism, metallic-like conductivity, power and energy storing aptitude [8,9]. The MXene in bare form obsessed low specific capacitance about 246 (F/g) in supercapacitors, but it improved its capacitive nature, significant specific surface area, hydrophilic nature, porous structure, negatively charged surfaces by recombination with other materials in nanocomposite form [10,11,12,13]. In energy storage applications i.e., in supercapacitors the transition metal oxides (TMOs) exhibited mixed spinal structure because of remarkable electrochemical properties were taken into consideration [14,15]. Hence metal oxide systems including metal chromite spinels, for instance MgCr_2_O_4_ [16] and CaCr_2_O_4_ [17]_,_ these exceptional spinels are verified to be high-performance electrode materials for electrochemical supercapacitors which may be taken either in single or in composite form with carbon-based materials for better results [18]. Among various silver-based compounds, (Ag_2_CrO_4_) nanoparticles in which crystallization occurred in orthorhombic form [19], Ag_2_CrO_4_ in composite form such as (Ag_2_CrO_4_/GO) composites [20], TiO_2_/Ag_2_CrO_4_ nanocomposites [21], Ag_2_CrO_4_/g-C_3_N_4_, RuO_2_-MXene along with silver, Co_3_O_4_/MXene, MXene/Ag, TiO_2_/Ti_3_C_2_, lanthanum and manganese co-doped bismuth ferrite/Ti_3_C_2_, MXene (Ti_3_C_2_T_x_)/Ag NWs (silver nanowires) had been synthesized in the past for various energy storage purposes [22,23,24,25,26,27,28,29,30]. The most recent work done on copper-chromite/graphene-oxide nanocomposite for electrode fabrication explored new energy tools had been accepted in my own collaboration [31]. However, there was no earlier work done in this field until now and thus this paper can be claimed as a novel work. The affordable wet chemical co-precipitation sonicated-assisted mechanical method of mixing was practiced during synthesis process of MXene/Ag_2_CrO_4_ nanocomposite which made it fit candidate for supercapacitive applications. Ag_2_CrO_4_ nanoparticles adding up with MXene sheets enhanced the capacitive properties of MXene by generating a course to electron transfer that led to unique surface contact within MXene sheets. We put forward the challenges and perspectives for the future progress of the MXene/Ag_2_CrO_4_ nanocomposite.

## 2. Materials and Methods

### 2.1. Chemical and Reagents

Silver nitrate pentahydrate (99.0%), chromium nitrate monohydrate (99%), 1, 2 ethanediol (99.8% pure) and powder form of tin was used. Here acetic acid (99.9%) acting as a catalytic agent, ethylene glycol (99.5%) was engrossed in it both for solution and reduction. Additionally, hydrofluoric acid known as HF (39%) was applied as etching agent in MAX (Ti_3_AlC_2_) powder. Deionized water (DI) was used as a solvent. The chemicals collected from sigma Aldrich company were used.

### 2.2. Ag_2_CrO_4_ Nanoparticles Synthesis

In order to synthesize Ag_2_CrO_4_ nanoparticles, the wet chemical sol-gel method was applied. In this approach, 4 g of silver nitrate and 3 g of chromium nitrate solution was prepared in 50 mL of DI water accomplished by adding up of citric acid powder in 2:1 ratio. The aqueous solution undergone continuous stirring at 70 °C until the required homogeneous solution obtained. After viscous gel development, stirring had been stopped. In order to achieve main product, solution was positioned at oven adjusted at 700 °C for three hours and then further calcination at 600 °C in the furnace was performed. At the end, powder form of the sample grounded in agate motor to get homogeneous fine powder. The chemical formula of Ag_2_CrO_4_ was explained by chemical Equations (1) and (2) given below [32].

Ag = Ag^+^ + e^-^ (1)

2Ag^+^ + CrO_4_^−2^ = Ag_2_CrO_4_ (2)

The obtained powder of Ag_2_CrO_4_ nanoparticles was used to synthesize nanocomposite of MXene/Ag_2_CrO_4_.

### 2.3. Synthesis of MXene (Ti_3_C_2_T_x_)

MXene was synthesized using the conventional method. First, 10 g of formerly prepared MAX powder were taken in a teflon bottle with 200 mL (39%) intense HF to synthesize Ti_3_C_2_T_x_ (MXene). Hydrofluoric acid (HF) and MAX powder was homogeneously blended by constant stirring for 36 h, at room temperature. Later, the hot plate was removed, and the solution was placed to cool down for 12 h. Moreover, the mixture was again stirred for 12 h. At the end, the resultant solution was rinsed several times by using deionized (DI) water followed by vacuum filtration. By drying the solution at 60 °C for 12 h, the etched MXene obtained is used for assembling of nanocomposite [33].

### 2.4. Synthesis of MXene/Ag_2_CrO_4_ Nanocomposite

The easily available wet chemical method, namely the co-precipitation method, was used to synthesize nanocomposite of MXene/Ag_2_CrO_4_ in which the solution of MXene prepared in 200 mL DI water by taking 200 mg of Ti_3_C_2_T_x_ (MXene) under sonication for 10 min. At the same time already prepared Ag_2_CrO_4_ nanoparticles were assorted in 100 mL acetic acid and 100 mL ethylene glycol in a stoichiometric proportion of 1:1 with 0.01 M (morality). Sonication of MXene solution was performed at 3500 rpm for 120 min at 60 °C to obtain homogeneous sample. Then, both solutions were thoroughly mixed by continuous stirring at 80 °C for 1 hr. After that the settled solution was washed many times with DI water unless neutral solution was obtained. An oven was used at 70 °C for 24 h until it is completely dried. Obtained nano-powder became homogenous using an agate motor.

## 3. Results and Discussion

### 3.1. X-ray Diffraction (XRD) Analysis

**Figure 1:**
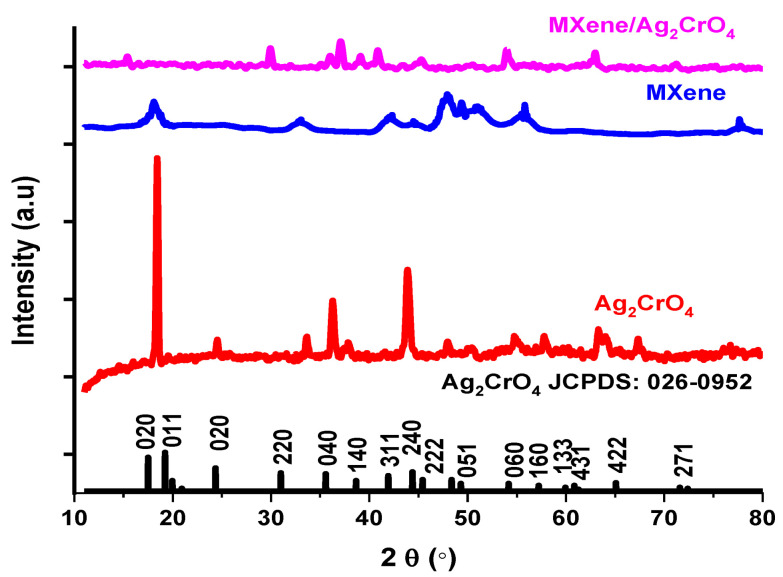
XRD patterns of Ag_2_CrO_4_, MXene and MXene/Ag_2_CrO_4_ nanocomposite.

The structure of the resultant sample was analyzed by X-Ray diffraction (XRD) technique with monochromatic wavelength λ (1.5 Å) in which corresponding (hkl) values were assigned approximately to all peaks. In Figure 1, the XRD pattern of bare Ag_2_CrO_4_ nanoparticles representing an orthorhombic structure with the JCPDS no. 026-0952 in which Bragg diffraction peaks appeared at 2θ = 24.45°, 33.65°, 36.34°, 37.92°, 43.82°, 47.97°, 50.36°, 54.95°, 57.91°, 63.42°, 67.18° confirming successful synthesis of Ag_2_CrO_4_ nanoparticles [34]. The prominently solid and high-pitched peaks proved pure and well-crystalline Ag_2_CrO_4_ collected by the stated process [35]. The characteristic peaks of MAX at 9.5° and 19.2° angles with (002) and (004) planes transferred towards left due to etched aluminum (Al) peaks resulting an increase in the spacing between sheets of resulting etched MAX powder so-called MXene (Ti_3_C_2_T_x_) [36]. In MXene/Ag_2_CrO_4_ nanocomposite all the peaks shifted towards lower angles with low intensity, certified an increase in inter-planar spacing from 4.7 Å to 6.2 Å of MXene/Ag_2_CrO_4_ nanocomposite which step up the conductivity [37].

From XRD data [38] the average crystallite size of MXene/Ag_2_CrO_4_ nanocomposite was calculated by Debye-Scherrer Equation (3) given below.

D = K λ/(β Cosθ)(3)

In general, crystalline size D in nm, X-ray wavelength λ is 0.15 nm, θ is the Bragg’s angle in radian, β full width half maximum of diffracted beams. The average crystallite size of MXene/Ag_2_CrO_4_ nanocomposite is 14 nm analogous to other MXene composite [39,40]. The presence of characteristic peaks of Ag_2_CrO_4_ and MXene in the nanocomposite sample is an indication of the successful development of MXene/Ag_2_CrO_4_ nanocomposite.

### 3.2. The Scanned Electronic Microscopic Analysis

**Figure 2:**
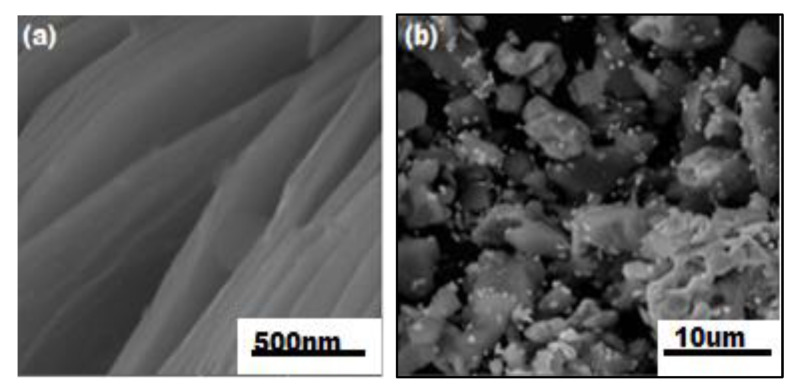
Images obtained from SEM of (**a**) MXene (Ti_3_C_2_T_x_), (**b**) MXene/Ag_2_CrO_4_ nanocomposite.

**Figure 3:**
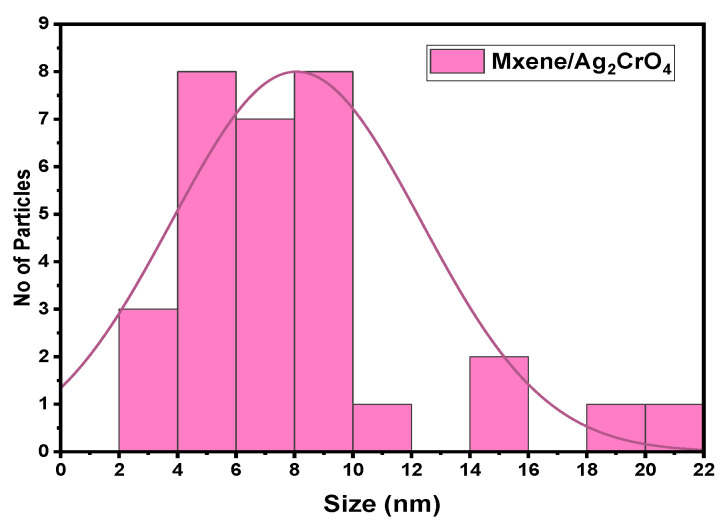
Particle size distribution histogram determined by SEM images.

The scanned electron microscopic (SEM) analysis of the synthesized sample explained the surface morphology of MXene/Ag_2_CrO_4_ nanocomposite. The purpose was to see how MXene and Ag_2_CrO_4_ nanoparticles coordinated with each other, including the even and continuous layered form of MXene with sharp edges gained after selective etching of aluminum (Al) layer by HF etching method as shown in Figure 2a. The SEM images of the Ti_3_C_2_T_x_/Ag_2_CrO_4_ nanocomposite sample are shown in Figure 2b in which nanoparticles of Ag_2_CrO_4_ adorned the surface of Ti_3_C_2_T_x_ in random pattern forming coagulated structure and explored huge clusters of the nanoparticles. Hence, only some grains scattered on layers of MXene. The number of nanoparticles nucleated on the surface of MXene engraved pores caused more storage capacity [41,42,43,44]. The average diameter of Ag_2_CrO_4_ nanoparticles is 75 nm reported in [32], here 3.67 nm is the grain size of MXene/Ag_2_CrO_4_ nanocomposite calculated by using image J. software. Here, clearly, it can be seen Ag_2_CrO_4_ nanoparticles in the MXene/Ag_2_CrO_4_ nanocomposite were reduced suggestively and closely occupied the MXene sheets. Furthermore, grain size distribution histogram shown in Figure 3 summarizing discrete or continuous data on an interval scale, respectively.

### 3.3. Energy Dispersive X-ray Spectroscopy (EDX)

**Figure 4:**
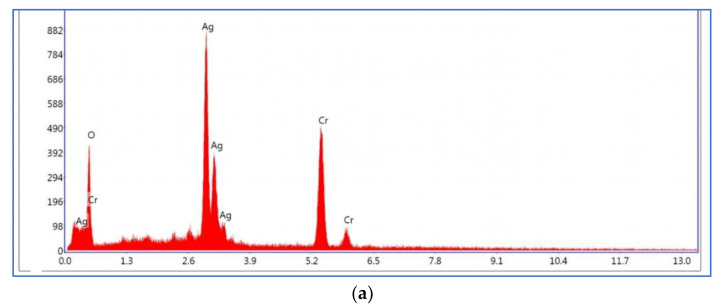

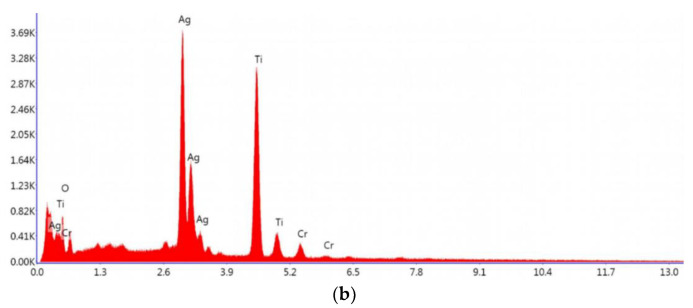
Energy dispersive spectra of (**a**) Ag_2_CrO_4_ nanoparticles (**b**) MXene/Ag_2_CrO_4_ nanocomposite.

The spectroscopy of energy dispersion analysis of Ag_2_CrO_4_ nanoparticles and MXene/Ag_2_CrO_4_ powder are exhibited in Figure 4a,b, respectively, where not only the Ag, Cr and Ti signals seemed but also the O signal appeared due to oxidation of MXene concerned with some functional groups. This provided the proof of perfect synthesis of current nanocomposite [45]. The elements presented in spectra as per EDX analysis according to weight percentage is confirmation of the ideal synthesis of the required MXene/Ag_2_CrO_4_ nanocomposite as shown in Table 1.

### 3.4. Raman Spectroscopy

Here, Raman spectroscopy has been employed to illustrate extremely responsive composition of the material structure having incredibility and a more mechanically important spectroscopic technique to probe the dynamic vibrational phonons of Ti_3_C_2_T_x_/Ag_2_CrO_4_ nanocomposite [46]. The Raman spectra of MXene (Ti_3_C_2_T_x_) was determined at 155 cm^−1^ showing a vibrational band of anatase phase of TiO_2_ [47]. Phonons (lattice vibrations) at the interface of MXene and traces of transition metal oxides were handled by Raman spectroscopy. Two main causes of lattice viberations in MXene based materials one, surface functional groups involved stimulating pseudocapacitance and the other, exchanging of ion gave rise to storing charge leading to a high capacitance of MXene/Ag_2_CrO_4_ nanocomposite in acidic solution [48,49]. 

**Figure 5:**
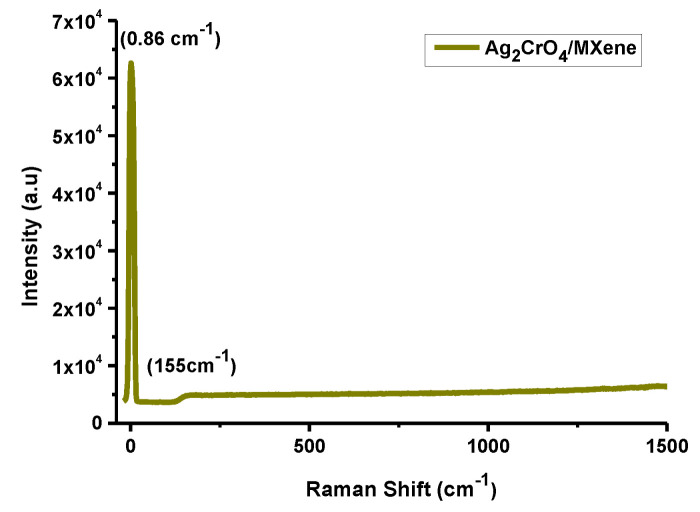
Raman spectra of MXene/Ag_2_CrO_4_ nanocomposite.

Raman spectroscopy of MXene/Ag_2_CrO_4_ nanocomposite noted at wavelength 532 nm and power 150 mW showing a characteristic peak at definite position 0.86 cm^−1^ with a remarkable intensity confirmed the occurrence of the prepared nanocomposite mostly due to the existence of functional groups involed [50] as shown in Figure 5.

### 3.5. Photoluminescence (PL) Spectroscopy

**Figure 6:**
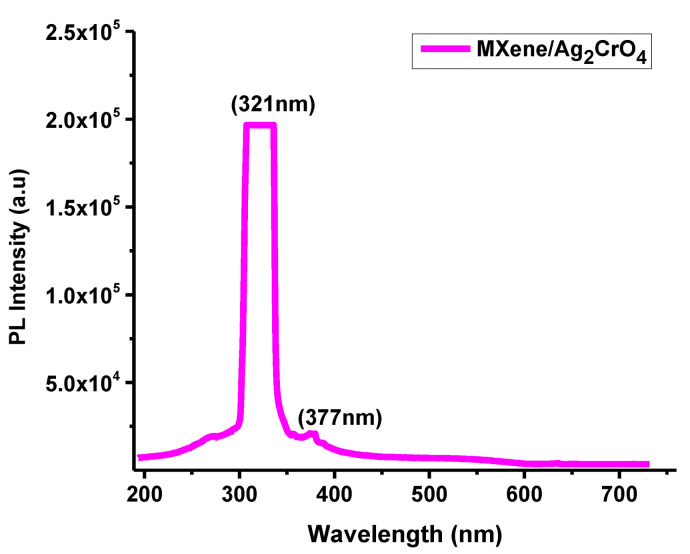
PL spectrum of Ti_3_C_2_T_x_/Ag_2_CrO_4_ nanocomposite.

The optical spectra of MXene/Ag_2_CrO_4_ nanocomposites were explained by using 325 nm wavelength of He-Cd laser at room temperature with 40 MW power. At 300 nm wavelength, the optical band gap 3.86 eV calculated in the visible region is indication of the allocation of nanoparticles clearly seen in Figure 6. The photoluminescence (PL) spectra of Ti_3_C_2_T_x_/Ag_2_CrO_4_ nanocomposite explored high intensity emission peak at 321 nm which was mainly due to electron-hole pair recombination of sp^2^ hybridized carbon atoms [51,52]. Due to defects in the structure of Ag_2_CrO_4_ the photoluminescence emission properties were possible at room temperature [34]. The recommended speed of charges transported by light irradiation effect on the Ti_3_C_2_T_x_/Ag_2_CrO_4_ nanocomposite in which valence band (VB) negative charges near to the ground skip to the conducting band (CB) due to complex photoluminescence scheme. When light was projected, positive and negative charges in aqueous medium coupled to produce radicals on the exterior of the Ti_3_C_2_T_x_/Ag_2_CrO_4_ nanocomposite [53].

### 3.6. Electrochemical Analysis

In order to perform the electrochemical analysis at Gamry potentiostat interface 1000, a three electrode assembly was taken where platinum wire, glassy carbon electrode (GCE) and Ag/AgCl were used as counter, working and reference electrodes, respectively [54,55]. The working electrode was rinsed many times using an alumina slurry and ethanol prior to production of synthesis material. To fabricate GCE, 0.25 g powder of electrode material was used with 2 µL of 5% nafion solution on glassy carbon electrode [56]. The functional electrode underwent drying in an oven at 50 °C for 20 min.

#### 3.6.1. Electrochemical Impedance Spectroscopy (EIS)

**Figure 7:**
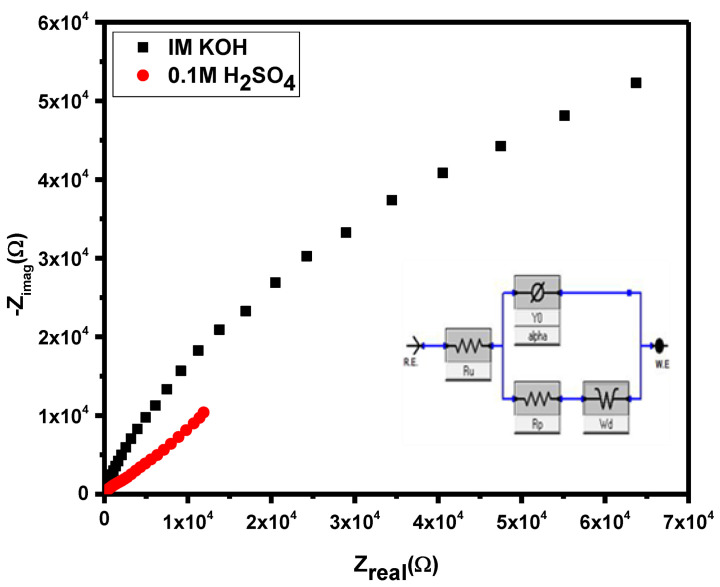
Nyquist plots of Ti_3_C_2_ T_X_/Ag_2_CrO_4_ in 1 M KOH and 0.1 M H_2_SO_4._.

The electrochemical impedance spectroscopy (EIS) was adopted to study the dependence of capacitance of supercapacitors on the applied power in which an alternating current voltage of 0.5 V and zero direct current voltage was utilized and current passes through electrode (metal or semiconductors) at working position [57]. The electron transferred properties of Ti_3_C_2_T_x_/Ag_2_CrO_4_ were studied by using EIS. The Nyquist plots drawn for Ti_3_C_2_T_x_/Ag_2_CrO_4_ in 0.1 M H_2_SO_4_ and 1 M KOH were displayed in Figure 7, also concerned EIS parameters were given in Table 2. The differences in electrochemical behavior of the as-synthesized electrocatalysts depend upon the relative feasibility of electron transfer. Low charge transfer resistance R_p_ due to elevated conduction, facilitated more electrons in the electrode surface and the current electrocatalysts showed a low R_p_ value [58] with high conductivity in acidic media, hence a higher specific capacitance C_sp_ value was achieved. The nature of the electrodes exhibited no influence on the solution resistance (Ru) and Warburg resistance (Rw) because these are features of the electrolyte and diffusion of electroactive specie that are common in all observations. However, (R_p_) and phase constant element (CPE) are influenced by modification of electrodes, as they are associated with conductive properties of the active material. Here α represents surface roughness factor and its value varies from 0 to 1. Herein, currently modified electrode system has α value 0.85 and 0.89 revealing that catalysts depicted enough surface roughness. The electron-transfer rate constant K_app_ (cm s^−1^) for planned catalysts was deliberated using the following Equation (4) [59]. Moreover, the fitted EIS model i.e., CPE with the diffusion model has been represented in the inset of Nyquist plots in Figure 7.

k_app_ = RT/F^2^·R_p_·C (4)

Here, F served as the Faraday constant, C corresponds to amount of analyte and R is the universal constant in SI units.

The poorer K_app_ in 1 M KOH aqueous electrolyte solution corresponds to relatively lower electron conductivity as compared to acidic electrolyte.

#### 3.6.2. Electrochemical Active Surface Area (ECSA) Analysis

**Figure 8:**
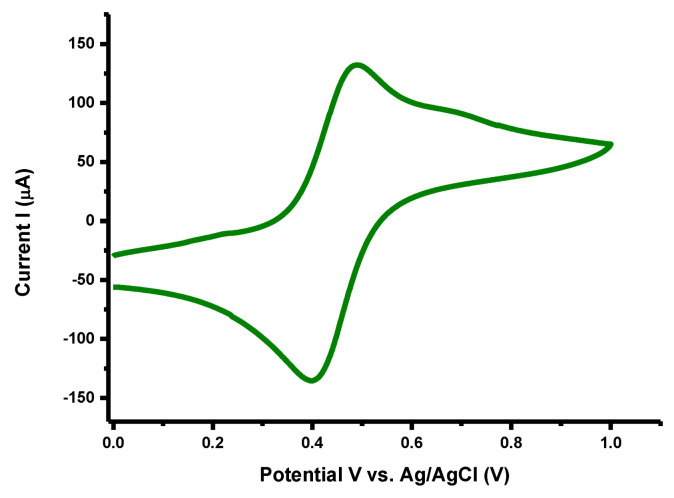
Cyclic voltammogram Fe^2+^/Fe^3+^ redox couple on Ti_3_C_2_T_x_/Ag_2_CrO_4_ modified GCE in 5 mMK_4_[Fe (CN)_6_]) + 3M KCl at 100 mVs^-1^.

The electrochemically active surface area (ECSA) is an important performance indicator of a catalyst in any electrochemical reaction and for this reason cyclic voltammograms of all prepared electrocatalysts were recorded in a standard redox solution of 5 mM potassium ferrocyanide (K_4_[Fe (CN)_6_]) and 3M potassium chloride (KCl) at 100 mVs^−1^ for ECSA inference [60]. Peak current (i_p_) increment in the CV profile correspond to a reversible one-electron transfer process using the synthesized nanocomposite as modified electrodes in K_4_[Fe (CN)_6_] electrolyte. This observation of a reversible CV profile was used to point out the oxidation and reduction methods by an overall redox process as shown in Figure 8. The ECSA of electrode was calculated by applying the Randles-Sevcik Equation (5) [61].

i_p_ = 2.69 × 10^5^·n^3/2^ ·A·D^1/2^ ·υ^1/2^ ·C (5)

Here i_p_ is the peak current, n is the count of transferred electrons, A is the electrochemical active surface area (cm^2^), D corresponds to the diffusion co-efficient, υ represents the scan rate (Vs^−1^), C is analytic amount [62]. With a peak current value of 132 μA, the ECSA of observed electrode is 0.04 cm^2^ that referred to efficient capacitive performance of the electrode material.

#### 3.6.3. Electrochemical Investigations

The electrochemical performances of Ti_3_C_2_T_x_/Ag_2_CrO_4_ nanocomposite were evaluated by cyclic voltammetry (CV) by varying the electrode potential between a working electrode and reference electrode in order to measure current flows between working and counter electrodes. By using the modified working electrodes in both acidic and basic electrolytes to analyze the electrode potential in both media, the acid electrolyte has the advantage in providing protons for as synthesized nanocomposite in cyclic voltammetry [63]. The capacitive behavior of the nanocomposite was observed in forward and reverse directions relative to the anodic peaks (oxidation) and cathodic peaks (reduction) which is the verification of surface redox reactions. The specific capacitance of working electrode Ti_3_C_2_T_x_/Ag_2_CrO_4_ nanocomposite was calculated using Equation (6).

C_sp_ = A/2 [mkV] (6)

where C_sp_ is specific capacitance in F/g, m is used for mass of electrode i.e., 0.25 mg, k represents the scan rate and A denotes integrated area under CV curve and V corresponds to potential window −0.2 V to 0.6 V. The estimated electrochemical capacitance parameters are summarized in Table 3.

**Figure 9:**
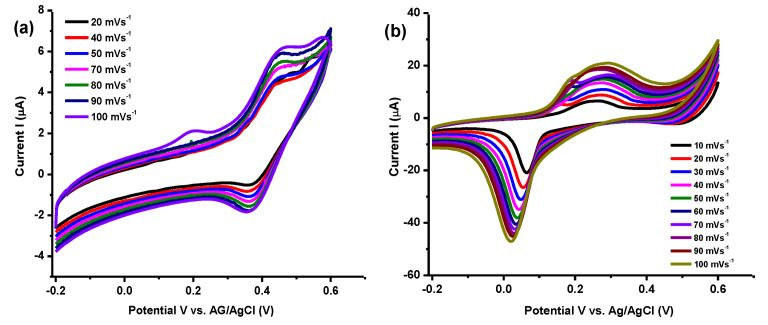
Voltametric profiles using Ti_3_C_2_T_x_/Ag_2_CrO_4_ electrode (**a**) in 1 M KOH (**b**) 0.1 M H_2_SO_4._.

Cyclic voltammetry demonstrated capacitive performance of Ti_3_C_2_T_x_/Ag_2_CrO_4_ electrode at different scan rate as shown in Figure 9. The highest C_sp_ = 525 F/g was attained at 10 mVs^−1^ in 0.1M H_2_SO_4_. Clearly, it can be seen from Table 3 that the specific capacitance and scan rate are inversely related. With an increase in scan rates, capacitance will be low owing to low charge storage ability of electrode material [64]. Enhanced specific capacitance with small area utilization by ions of electrolyte at a low scan rate is important to note down [65]. Synthesized Ti_3_C_2_T_x_/Ag_2_CrO_4_ nanocomposite exhibits comparatively improved capacitance output even at lower concentration of acidic electrolyte. Comparison of Ti_3_C_2_T_x_/Ag_2_CrO_4_ nanocomposite with other nanocomposites shown in Table 4.

## 4. Conclusions

MXene (Ti_3_C_2_T_x_) based silver-chromite nanocomposite special treatment particularly in the field of energy storage application is reported. XRD showed enhanced inter-planar spacing from 4.7 Å to 6.2 Å. SEM images revealed silver chromite nanoparticles attachment to MXene sheets whereas EDX confirmed the presence of silver chromite within the nanocomposite. Raman spectroscopy and photoluminescence revealed functional groups’ attachment and a band gap value of about 3.86 eV. MXene/Ag_2_CrO_4_ nanocomposite-based electrode in 0.1M H_2_SO_4_ electrolyte have 525 F/g capacitance at a scan rate of 10 mVs^−1^ instead of its lower value of 75 F/g at 20 mVs^−1^ in case of 1M KOH. Thus, pseudocapacitive behavior in the acidic media gives maximum charge storage in the case of the Ti_3_C_2_T_x_/Ag_2_CrO_4_ electrode, as compared to basic media. MXene type materials in nanocomposite form with significant capacitance in the near panorama give strategy to suggest more search. Here specific capacity of Ti_3_C_2_T_x_/Ag_2_CrO_4_ electrode faraway from ideal value, for this reason there is need for progress in the instruction about surface functional groups.

## Figures and Tables

**Table 1:** Estimated elemental composition of MXene/Ag_2_CrO_4_ nanocomposite.

Elements	Shell	Weight (%)
Oxygen	K	14.10
Silver	K	43.41
Titanium	K	39.04
Chromium	K	3.04

**Table 2:** Electrochemical parameters estimated from EIS analysis Ti_3_C_2_T_x_/Ag_2_CrO_4_ modified electrode.

Electrolyte	R_u_ (Ω)	R_p_ (kΩ)	CPE (µF)	Alpha	R_W_ (µΩ)	K_app_ (10^−8^ cm s^−1^)
1M KOH	17.52	52.60	7.90	0.85	19.63	0.03
0.1M H_2_SO_4_	7.80	1.35e^−6^	0.94	0.89	92.03	3953

**Table 3:** Specific capacitance measurements from cyclic voltametric measurements.

Scan Rate (m Vs^−1^)	Specific Capacitance (F/g) in 1 M KOH	Specific Capacitance (F/g) in 0.1 M H_2_SO_4_
10	-	525
20	75	348
40	40	239
70	29	176
80	28	161
100	26	148

**Table 4:** Comparison of the specific capacitance with earlier MXene based nanocomposite electrodes.

Electrode	Electrolyte	Scan Rate (mVs^−1^)	Capacitance (F/g)	References
Ti_3_C_2_T_x_/Ag_2_CrO_4_	0.1M H_2_SO_4_	10	525	This work
Ti_3_C_2_T_x_ Aerogels	3M H_2_SO_4_	10	438	[66]
Ti_3_C_2_T_x_ ion gel	Ionic liquid	20	70	[67]
Ti_3_C_2_Tx/PPy	1M H_2_SO_4_	5	416	[68]
Ti_3_C_2_Tx/PPy nanoparticles	1M Na_2_SO_4_	2	184.36	[69]

## Data Availability

All the data is contained in the manuscript.
